# Flowering in Space: Transcriptional Insights Into the Crosstalk Between Aging, Gibberellin, and Sugar Pathways to Modulate Flowering in Space

**DOI:** 10.1111/ppl.70460

**Published:** 2025-08-15

**Authors:** Zeeshan Nasim, Nouroz Karim, Ji Hoon Ahn

**Affiliations:** ^1^ Department of Life Sciences Korea University Seoul Korea

**Keywords:** arabidopsis, flowering, spaceflight, SPLs

## Abstract

Spaceflight presents a unique environment that affects plant development, including flowering time. Using transcriptomic data of Arabidopsis seedlings grown aboard the International Space Station, we found that spaceflight conditions modulate flowering through coordinated hormonal and genetic pathways. Elevated expression of gibberellin biosynthesis genes suggests increased GAs accumulation, which likely promote SPL transcription factor expression and activity by degrading DELLA repressors, while altered sugar signaling represses miR156, contributing to the upregulation of SPLs. This cascade activates miR172, suppressing floral repressors and inducing integrator genes such as AGL24, ultimately triggering floral transition. Our findings identify SPLs as a central regulatory hub in spaceflight‐induced flowering and reveal how space conditions reprogram developmental signaling networks. Our findings can be important for advancing our understanding of plant adaptation beyond Earth and support the development of controlled flowering strategies in extraterrestrial agriculture. This work contributes to the broader goal of creating sustainable bioregenerative life support systems for long‐duration space missions.

Spaceflight presents a unique environment for plant growth, characterized by microgravity and altered radiation, which induce novel physiological and molecular responses in plants. As humanity advances toward long‐duration space missions and potential extraterrestrial colonization, understanding plant biology in space has become increasingly important. Plants are essential components of bioregenerative life support systems, providing oxygen, food, and psychological benefits for astronauts. However, the space environment presents previously uncharacterized challenges to plant developmental processes, particularly flowering. The transition to flowering is a critical developmental switch, and its precise regulation is essential for reproductive success. In Arabidopsis, a commonly used model plant, flowering is regulated by complex genetic networks comprising nearly 400 genes, which ensure the integration of several environmental cues into developmental reprogramming (Bouché et al. 2016). Among the various physiological processes affected by spaceflight, alterations in gene expression, especially of flowering genes, are of prime importance in understanding flowering and preparing for a potential future in space.

A pioneering study utilized transgenic *Arabidopsis* expressing *FLOWERING LOCUS T* (*FT*), the florigen, under a heat shock‐inducible promoter, allowing the remote induction of *FT* expression during spaceflight (Wang et al. 2022). *FT* induction in space promoted flowering even under non‐inductive short‐day conditions and resulted in reduced inflorescence stem elongation, compared to wild‐type plants. Transcriptome analyses revealed several photoperiod‐responsive genes to be affected by spaceflight, particularly those involved in post‐translational modification, such as protein phosphorylation (Wang et al. 2022). Transcriptome analyses of another study have shown reduced expression of *FT* and *SUPPRESSOR OF OVEREXPRESSION OF CONSTANS 1* (*SOC1*), two central floral integrators, in response to spaceflight, leading to delayed flowering (Xie et al. 2021). The circadian oscillator, which coordinates daily rhythms in gene expression, is a central integrator of environmental signals affecting flowering time (Shim et al. 2017). Spaceflight has also been shown to alter the expression of core circadian clock genes, including *LATE ELONGATED HYPOCOTYL 1* (*LHY*), *CIRCADIAN CLOCK ASSOCIATED 1* (*CCA1*), *GIGANTEA* (*GI*), and *EARLY FLOWERING 4* (*ELF4*), as well as photoperiod pathway components such as *CONSTANS* (*CO*) and *FT* (Wang et al. 2022). In space‐grown *Arabidopsis* seedlings, morning‐phased genes (*CCA1* and *LHY*) are upregulated, whereas evening‐phased genes (*ELF4 and GI*) are downregulated under both long‐day and short‐day photoperiods. These disruptions can interfere with the precise timing of *FT* expression, the florigen and a key integrator of flowering signals, potentially leading to altered flowering times in space (Wang et al. 2022). These transcriptional changes highlight the sensitivity of flowering regulatory networks to spaceflight‐associated gravitational and environmental cues and suggest that the circadian oscillator and photoperiodic pathways are key modulators of flowering time under these conditions.

Despite these insights, a comprehensive global‐scale analysis of the regulation of flowering genes in response to spaceflight remains lacking. To do so, we utilized the publicly available RNA‐seq data (GSE148914) to assess the transcriptional response of all known flowering‐related genes in wild‐type Arabidopsis (Columbia accession) seedlings exposed to spaceflight during the SpaceX‐CRS12 mission to the International Space Station (Angelos et al. 2021). The data were analyzed using the HISAT2‐Cufflinks pipeline (Trapnell et al. 2012; Kim et al. 2019). Differentially expressed genes (DEGs) were defined as genes with a fold‐change of two or more and a total fragments per kilobase of transcript per million (FPKM) value of two or more. The DEGs were then cross‐referenced with the FLOR‐ID database (Bouché et al. 2016) which contains all currently annotated flowering genes. Our analysis identified 30 flowering genes that were downregulated and 32 genes that were upregulated in response to spaceflight compared to ground controls (Figure 1A). These DEGs were then categorized into various flowering regulatory pathways. Our analyses showed differential expression of several photoperiod pathway genes (Figure 1B), consistent with previous findings (Wang et al. 2022). *CONSTANS* (*CO*), encoding a key modulator of the photoperiod pathway and positive regulator of *FT* (Suárez‐López et al. 2001), which was previously reported to be upregulated in response to spaceflight (Wang et al. 2022), also showed a 3.1‐fold increase in our analysis. However, a downstream target gene of the photoperiodic pathway, *FT*, was not among the DEG between space‐grown and ground‐grown control seedlings (Figure 1B), suggesting that, under the tested conditions, the photoperiod pathway is unlikely to be the primary modulator of flowering in space. Notably, four members of the miR156 family (miR156a, c, d, and f) were significantly downregulated in response to spaceflight (Figure 1B). miR156a was downregulated by 4.7‐fold, miR156c by 6.9‐fold, while miR156d and miR156f exhibited a 39‐ and 2.4‐fold decrease, respectively (Figure 1C). Since miR156 acts as a negative regulator of *SQUAMOSA PROMOTER BINDING‐LIKE* (*SPL*) transcription factors (Yang et al. 2011; Schwarz et al. 2008), this downregulation is consistent with the observed upregulation of *SPL* genes. *SPL3* was upregulated by 4.6‐fold, while *SPL4*, *SPL5*, and *SPL9* transcript levels were increased by 19.4‐, 3.3‐, and 4.2‐fold, respectively (Figure 1D), suggesting that spaceflight affects the aging pathway.

**FIGURE 1:**
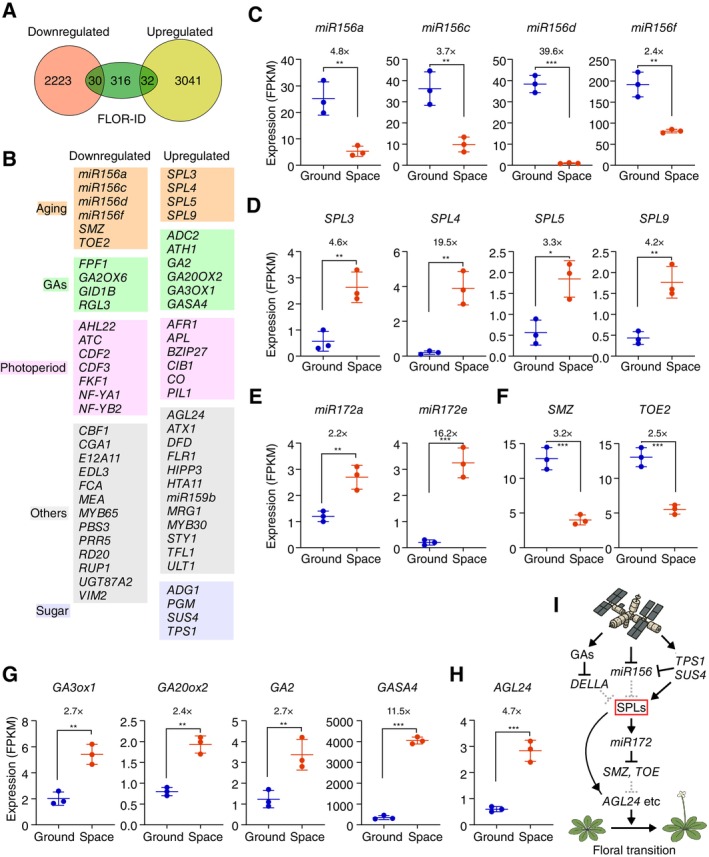
Spaceflight modulates the expression of genes in Gibberellin (GA), aging, and sugar signaling pathways. (A) Identification of differentially expressed flowering time genes in response to spaceflight using publicly available RNA‐seq data (GSE148914), previously reported (Angelos et al. 2021). (B) Classification of genes into different flowering regulatory pathways. Colors represent different flowering pathways indicated on the left of the DEG classification table. (C–H) RNA‐seq‐derived expression levels of flowering genes. Y‐axis indicates expression values in Fragments Per Kilobase of transcript per Million mapped reads (FPKM). (C) miR156 family genes; (D) *SPL* genes; (E) miR172a and miR172e; (F) *SMZ* and *TOE2*; (G) GA biosynthesis and GA‐responsive genes; (H) *AGL24*, a floral integrator gene. (I) Proposed model of flowering time regulation in space. The values above the bars show the fold‐change in expression between spaceflight and ground samples. *p < 0.05; **p < 0.01; ***p ≤ 0.001.

In *Arabidopsis*, miR156 negatively regulates miR172 through its repression of *SPL* genes, forming the core of the age‐related flowering pathway (Hyun et al. 2017). Given the increase in *SPL* gene expression (Figure 1D), we hypothesized that miR172 levels would also be elevated, as SPL transcription factors are known to activate miR172 transcription (Wu et al. 2009). Indeed, miR172a and miR172e levels were upregulated by 2.5‐ and 16.2‐fold, respectively (Figure 1E). Consistent with this, the two key downstream targets of miR172, *SCHLAFMÜTZE* (*SMZ*) and *TARGET OF EARLY ACTIVATION TAGGED 2* (*TOE2*), two AP2‐like transcription factors that repress flowering (Hyun et al. 2017), were significantly downregulated by 3.2‐ and 2.5‐fold, respectively (Figure 1F). Collectively, these findings suggest that spaceflight modulates flowering time, at least in part, via the age‐regulated miR156–SPL–miR172 module.

Our transcriptome analyses also revealed several gibberellin (GA)‐related genes to be differentially expressed in response to spaceflight (Figure 1G). GAs are known to promote the expression and activity of SPL transcription factors by targeting DELLA (GAI/RGA/RGL family) proteins for degradation. DELLAs bind directly to SPLs and inhibit their transcriptional activation; elevated GA levels trigger DELLA degradation, thereby releasing SPLs to activate downstream targets such as miR172 and MADS‐box genes (Hyun et al. 2017). GA levels are tightly regulated by a number of activating and deactivating enzymes, including GIBBERELLIN 3‐OXIDASE 1 (GA3OX1), GIBBERELLIN 20‐OXIDASE 2 (GA20OX2), and GA REQUIRING 2 (GA2), all of which play important roles in GA biosynthesis (Bao et al. 2020). Our results showed significant upregulation of GA biosynthesis genes *GA3OX1*, *GA20OX*, and *GA2* with a 2.7‐, 2.4‐, and 2.7‐fold increase, respectively (Figure 1B,G), suggesting elevated GA biosynthesis and accumulation under spaceflight conditions. This is further supported by the strong induction of *GA‐STIMULATED ARABIDOPSIS 4* (*GASA4*), a GA‐responsive gene, which showed an 11.5‐fold increase in expression in spaceflight‐grown plants compared to ground‐grown control plants (Figure 1G). Furthermore, our transcriptome analyses also revealed upregulation of the floral meristem identity gene, *AGAMOUS‐LIKE 24* (*AGL24*), a key floral integrator involved in the transition from vegetative to reproductive growth (Bouché et al. 2016). *AGL24* was upregulated by 4.7‐fold in response to spaceflight (Figure 1H). Given that SPL transcription factors can activate *AGL24* (Wang et al. 2009; Bouché et al. 2016), this suggests that AGL24 serves as a downstream effector of spaceflight‐induced transcriptional reprogramming and may contribute to spaceflight‐modulated floral transition.

Alongside GAs, sugar signaling is also tightly integrated with the aging pathway to regulate flowering in Arabidopsis. TREHALOSE‐6‐PHOSPHATE SYNTHASE 1 (TPS1) catalyzes the formation of trehalose‐6‐phosphate (T6P), a signaling molecule that reflects cellular sucrose status and represses the expression of miR156. TPS1‐deficient plants exhibit delayed flowering, elevated levels of miR156, and correspondingly reduced expression of *SPL* genes (Wahl et al. 2013). Our analyses showed downregulation of *TPS1* and *SUCROSE SYNTHASE 4* (*SUS4*), key components of the sugar pathway, which may contribute to the reduced miR156 expression and derepression of *SPL*s in response to spaceflight (Figure 1B–D). However, further work is required to genetically validate the direct involvement of *SUS4* in regulating the miR156–SPL module.

In conclusion, we propose that spaceflight conditions influence flowering time through coordinated regulation of hormonal and developmental signaling pathways. Elevated GA levels in space promote the degradation of DELLA repressors, thereby enhancing SPL activity. Furthermore, spaceflight represses miR156, possibly via altered sugar signaling involving TPS1 and SUS4, further promoting SPL function. Active SPLs then induce miR172, which suppresses floral repressor genes like *SMZ* and *TOE*, leading to the induction of integrator genes such as *AGL24*, which triggers floral transition (Figure 1I). This model highlights the convergence of GA signaling, sugar signaling, and the age‐dependent miR156–SPL module, revealing a mechanistic crosstalk wherein hormonal cues modulate intrinsic developmental pathways to fine‐tune flowering in response to spaceflight conditions. While our study provides valuable insights into the transcriptional changes underlying flowering regulation in spaceflight, it may be limited by the specific growth conditions used. Standardized monitoring systems and multi‐omics approaches will be essential for deeper mechanistic insights. Expanding research across species and incorporating robust spaceflight simulations will be critical for advancing plant adaptation strategies in space agriculture.

## Author Contributions

Z.N. conceived the research direction; Z.N. performed most of the experiments; Z.N. performed bioinformatics analyses; Z.N., N.K., and J.H.A. interpreted data and wrote the manuscript.

## Data Availability

All data supporting the findings of this study are available within the article. The RNA‐seq data used in this work were previously published (Angelos et al. 2021) and are available under the accession number GSE148914.
